# Pedunculated Epipericardial Fat Torsion: A Subtle Clinical and Radiological Mimicker

**DOI:** 10.5334/jbsr.2748

**Published:** 2022-05-04

**Authors:** Baptiste A. Vande Berg, Loes Tanghe, Adriana Dubbeldam

**Affiliations:** 1 UZ Leuven, BE

**Keywords:** Epipericardial fat necrosis, mediastinal fat necrosis, acute chest pain, pleuritic chest pain, pulmonary embolism

## Abstract

**Teaching Point:** Epipericardial fat torsion is a little-known and uncommon condition with sometimes subtle findings making general awareness essential for detection and for confident diagnosis.

## Case History

**Figure 1:**
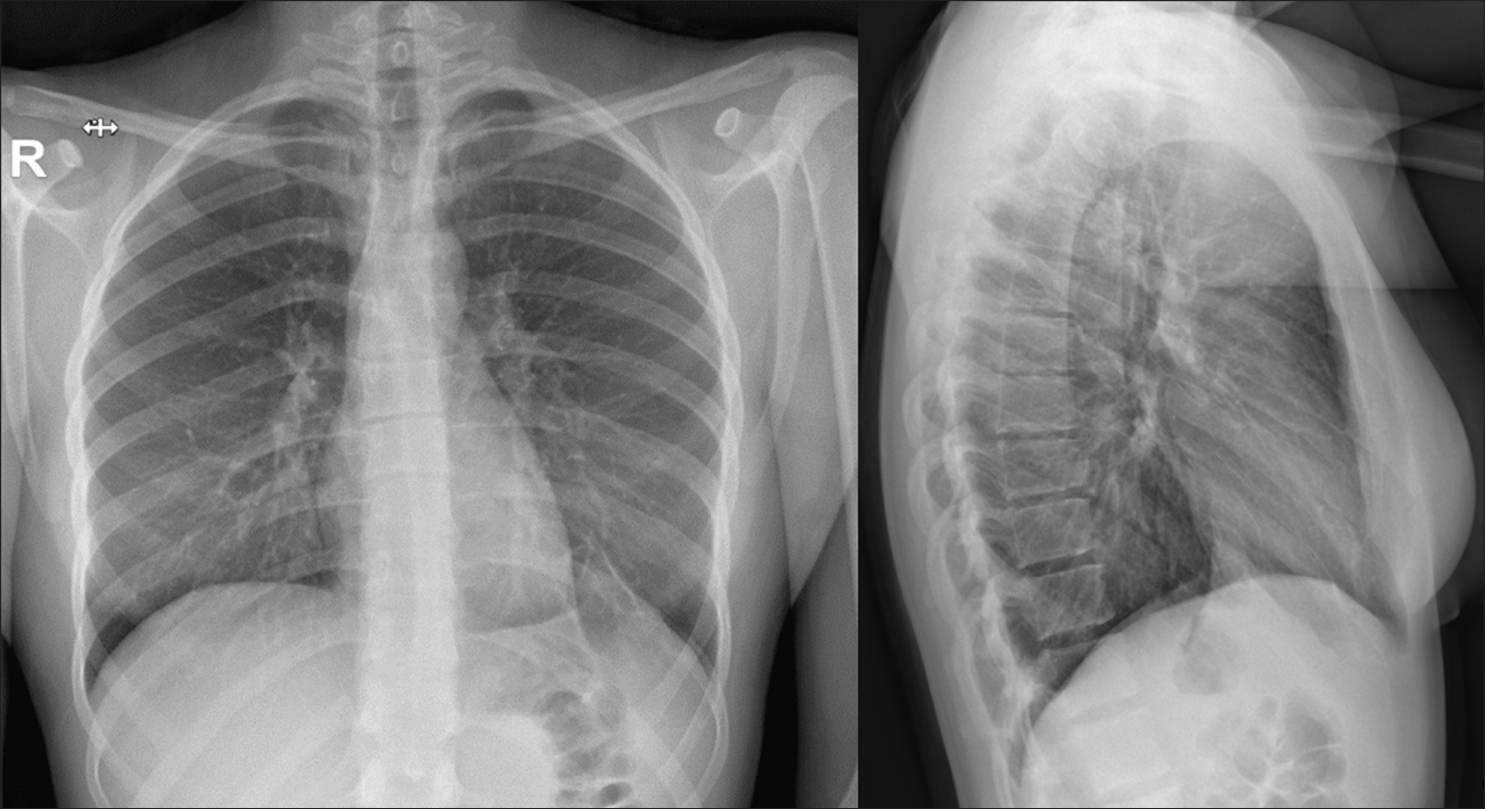


**Figure 2:**
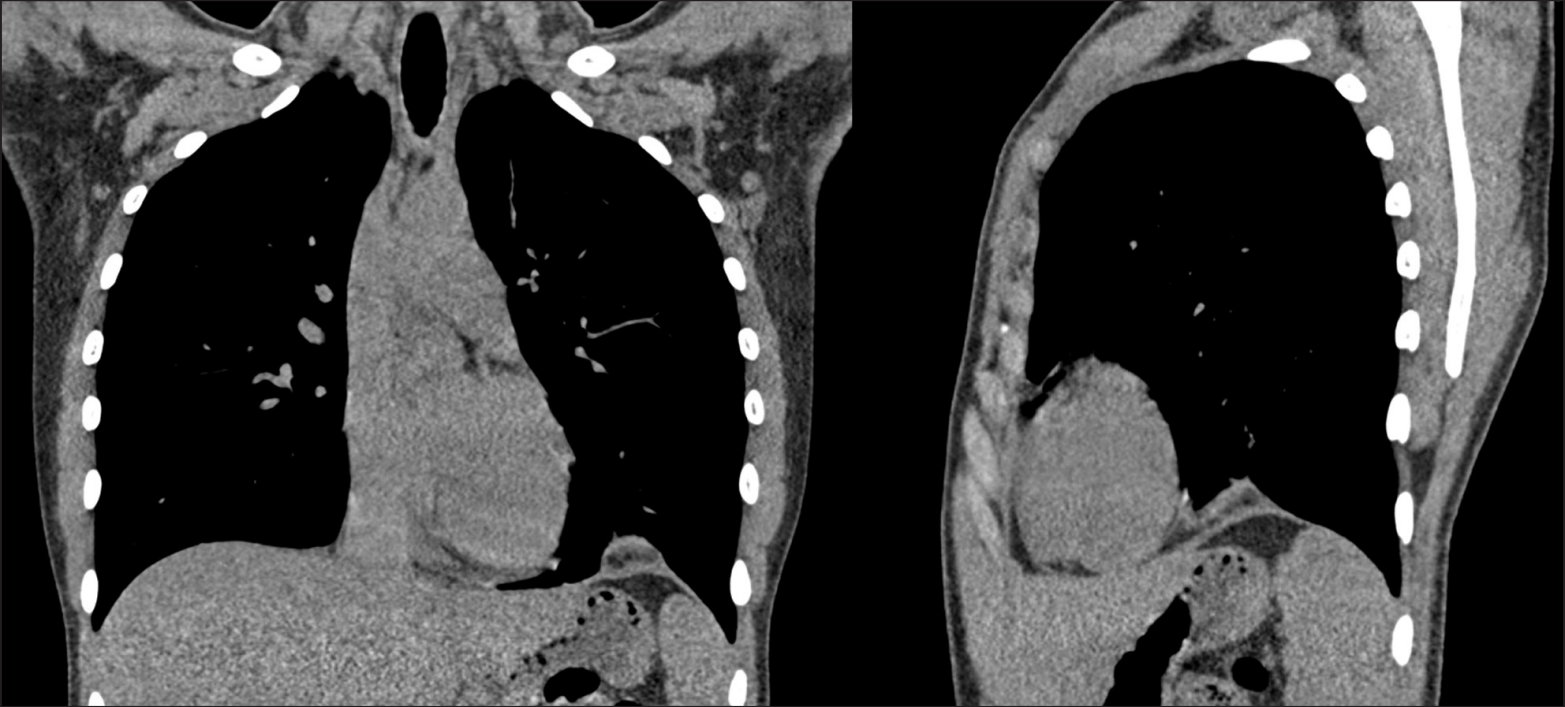


**Figure 3:**
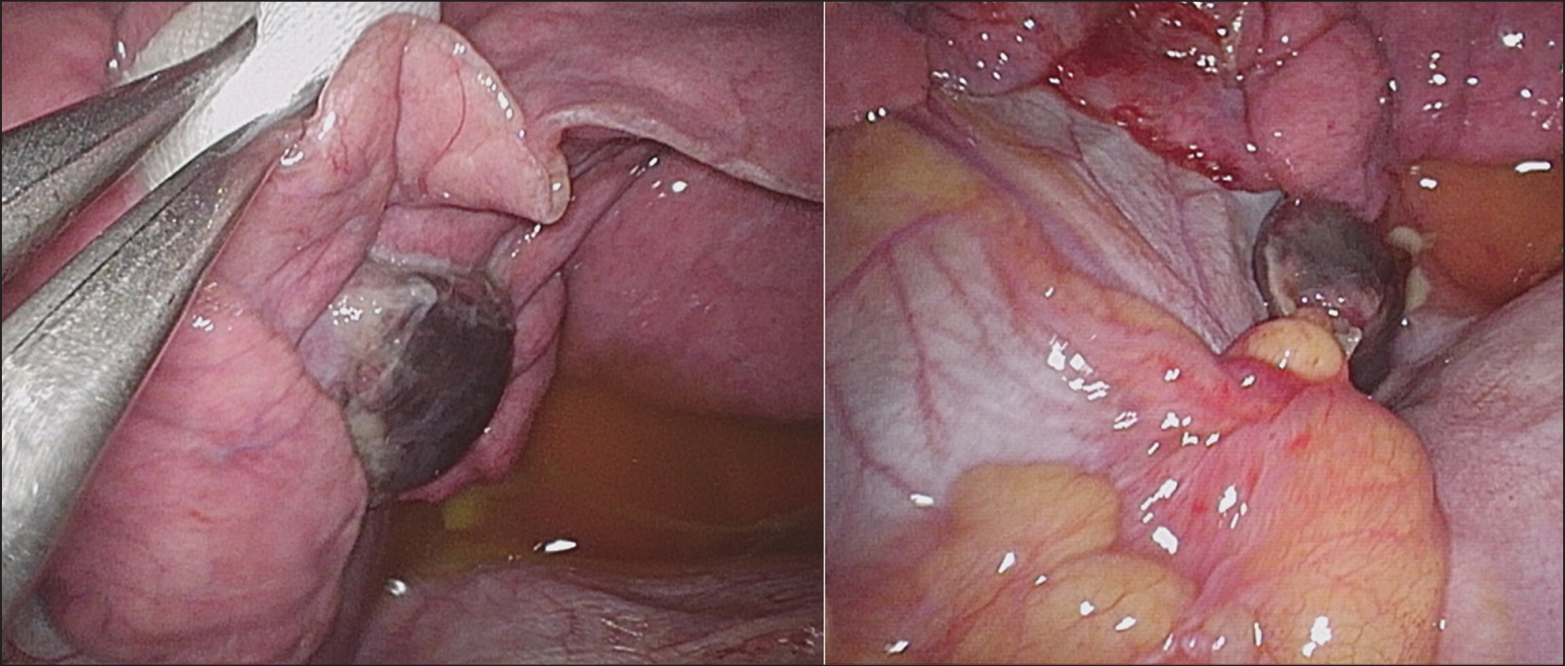


A 21-year-old woman presented to the emergency department with acute left-sided stabbing chest pain with radiation to the left shoulder. The suddenly arising pain was related to inspiration and to supine position but remained absent during expiration. No significant prior medical history or medication intake was noted, and the patient reported no other symptoms. She denied leg swelling or pain, recent flight travel, or smoking. Physical examination and electrocardiogram were unremarkable. A chest radiograph depicted a non-specific left-sided diaphragmatic mass with tenting of the diaphragmatic cupola (***Figure 1***). Further laboratory blood tests could exclude pulmonary embolism and infectious disease with low D-dimer and absence of systemic inflammation. With this suspicious clinical picture and diaphragmatic tenting, a thoracic computed tomography (CT) without intravenous contrast was requested. The mass-like image on the chest X-ray corresponded to a low density (–80 Hounsfield units) nodular structure with a surrounding soft tissue attenuation rim on the left diaphragmatic dome with a diameter of 2 cm and adjacent lung atelectasis (***Figure 2***). A clear connection to the mediastinum (***Figure 2***) was demonstrated, which raised the suspicion of an epipericardial fat torsion. An indication for surgery was retained for definite diagnosis and pain relief. Surgery showed no diaphragmatic defect but revealed a twisted epipericardial fat pad with necrosis adhering to the diaphragmatic side of the left lower lobe (***Figure 3***). At histology, circumscribed adipose tissue with ischemic necrosis, granulation tissue, hemorrhage, and multinucleated giant cells were seen. The patient had an uneventful recovery and was able to leave the hospital three days later in good general condition.

## Comment

This case is an illustration of an atypical localization of an epipericardial fat torsion and necrosis. Clinically epipericardial fat necrosis presents with acute unilateral chest pain, more frequently left-sided, without significant biochemical abnormalities [1]. Imaging is indispensable crucial for diagnosis. At chest radiography, an aberrant pericardial contour may be seen in combination with a small pleural effusion. In this case, the epi-diaphragmatic localization of the fat pad mimics the “juxtaphrenic peak” and the “melting ice cube sign” as seen in, respectively, left lower lobe atelectasis and subacute pulmonary embolism. With cross-sectional imaging, the typical appearance is that of an ovoid shaped fat-containing lesion with surrounding inflammation and eventually a small pleural or pericardial effusion [1]. Differential diagnosis at CT includes herniated intra-abdominal fat through a diaphragm defect, thoracic lipoma, and liposarcoma. Standard treatment is conservative with pain relief by non-steroidal anti-inflammatory drugs. However, in some cases surgery is indicated for relief of uncontrollable pain or to confirm the diagnosis.
